# Alginate–Bentonite Encapsulation of Extremophillic Bacterial Consortia Enhances *Chenopodium quinoa* Tolerance to Metal Stress

**DOI:** 10.3390/microorganisms12102066

**Published:** 2024-10-15

**Authors:** Cesar Arriagada-Escamilla, Roxana Alvarado, Javier Ortiz, Reinaldo Campos-Vargas, Pablo Cornejo

**Affiliations:** 1 Laboratorio Biorremediación, Departamento de Ciencias Forestales, Facultad de Ciencias Agropecuarias y Medioambiente, Universidad de La Frontera, Temuco 4811230, Chile; roxanalvaradoab@gmail.com (R.A.); javier.ortiz@ufrontera.cl (J.O.); 2 Programa de Doctorado en Ciencias de Recursos Naturales, Universidad de La Frontera, Temuco 4811230, Chile; 3 Scientific and Technological Bioresource Nucleus (BIOREN), Universidad de La Frontera, Temuco 4811230, Chile; 4 Center for Postharvest Studies, Faculty of Agricultural Sciences, Universidad de Chile, Santiago 8820808, Chile; reinaldocampos@uchile.cl; 5 Centro Regional de Investigación e Innovación para la Sostenibilidad de la Agricultura y los Territorios Rurales, CERES, Pontificia Universidad Católica de Valparaíso, La Palma, Quillota 2260000, Chile; pcornejo@centroceres.cl

**Keywords:** bacterial storage, extremophilic bacteria, metal(loid)s biosorption, metal(loid)s toxicity alleviation

## Abstract

This study explores the encapsulation in alginate/bentonite beads of two metal(loid)-resistant bacterial consortia (consortium A: *Pseudomonas* sp. and *Bacillus* sp.; consortium B: *Pseudomonas* sp. and *Bacillus* sp.) from the Atacama Desert (northern Chile) and Antarctica, and their influence on physiological traits of *Chenopodium quinoa* growing in metal(loid)-contaminated soils. The metal(loid) sorption capacity of the consortia was determined. Bacteria were encapsulated using ionic gelation and were inoculated in soil of *C. quinoa*. The morphological variables, photosynthetic pigments, and lipid peroxidation in plants were evaluated. Consortium A showed a significantly higher biosorption capacity than consortium B, especially for As and Cu. The highest viability of consortia was achieved with matrices A1 (3% alginate and 2% bentonite) and A3 (3% alginate, 2% bentonite and 2.5% LB medium) at a drying temperature of 25 °C and storage at 4 °C. After 12 months, the highest viability was detected using matrix A1 with a concentration of 10^6^ CFU g^−1^. Further, a greenhouse experiment using these consortia in *C. quinoa* plants showed that, 90 days after inoculation, the morphological traits of both consortia improved. Chemical analysis of metal(loid) contents in the leaves indicated that consortium B reduced the absorption of Cu to 32.1 mg kg^−1^ and that of Mn to 171.9 mg kg^−1^. Encapsulation resulted in a significant increase in bacterial survival. This highlights the benefits of using encapsulated microbial consortia from extreme environments, stimulating the growth of *C. quinoa*, especially in soils with metal(loid) levels that can be a serious constraint for plant growth.

## 1. Introduction

*Chenopodium quinoa* Wild. (quinoa) has received significant global attention in recent years due to its nutritional benefits, being considered the only food in the plant kingdom that provides all essential amino acids and meets the human and animal nutrition standards according to the FAO [1,2]. Several studies evidenced the ability of quinoa to adapt to nutrient-poor environments, saline soils, and drought-stressed agroecosystems [3]. However, studies related to metal tolerance of quinoa warn of the potential danger of metal accumulation (Fe^3+^, Zn^2+^, Cu^2+^, Cd^2+^, Ni^2+^, Cr^3+^, Pb^2+^, and Cu^2+^) in leaves and seeds [4,5,6]. Likewise, the metal accumulation in soils compromises agriculture and food production [7], causing oxidative damage [8], interfering in the absorption and distribution of nutrients [9], and affecting the biosynthesis of photosynthetic pigments in the plants [8,10]. Moreover, the risk of metal(loid) accumulation was previously identified, even when present in low concentrations in the soil, making plants unfit for human consumption [6].

Metal(loid)s’ resistant bacteria with plant-growth-promoting traits (PGPBs) are known to play an essential role in managing metal(loid) stress, such as Pb(II), Ni(II), Cd(II), Cr(III), Hg(II), Cu(II), Zn(II), Co(II), and As(II) in plants; for example, some species of *Bacillus*, *Pseudomonas*, and *Streptomyces* in plants of *Spinacia oleracea*, *Helianthus annuus*, or *C. quinoa*, respectively [11,12,13]. Therefore, there is a notable opportunity to use efficient PGPBs as a sustainable tool and a promising method, as an alternative or complement to transgenic genotypes and synthetic fertilizers, due to their strong contribution to plant resistance [14]. Some plants, such as *C. quinoa*, have been described as a potential supplier of PGPBs that enhance their physiological plant performance [15]. Metal(loid)-tolerant microorganisms are ubiquitous in extreme environments, such as hyper-arid ecosystems, contaminated soils [16,17], or Antarctica, and can produce and release a variety of plant-protective metabolites, transforming them into non-bioavailable forms in soils, and thus reducing their uptake by plants [18] and enabling pH regulation [19]. These PGPBs can reduce the absorption or translocation of metal(loid)s from the plant to the shoots through processes such as bioaccumulation, biosorption, precipitation, complexation, and alkalinization [20]. However, these effects arise not only from plant–microbe interactions, but also from microbe–microbe interactions such as competition and antibiotic production, among others [21]. Therefore, mixed inoculants can provide excellent results in plant production and show potential to be used by farmers because of their greater flexibility in different environmental conditions [22].

The soil is commonly a hostile, heterogeneous, and unpredictable environment, where inoculated bacteria (bioinoculants) cannot easily establish themselves [23]. As a way to ameliorate the above conditions, mixed inoculants are applied, with the aim of promoting plant growth through the combination of different molecular mechanisms displayed by the PGPBs in a consortium [24]. In this sense, different members of a microbial consortium can develop specific and beneficial functions for the soil and plants, as well as exert indirect effects at the level of the rhizosphere and microbial communities [25]. Several studies demonstrate the effectiveness and benefits of microbial consortia. They generally stimulate growth and control diseases in plants while also mitigating specific abiotic and biotic stresses in crops [26]. Furthermore, combinations of the same species (different strains) show beneficial effects, being considered a consortium [27]. For example, Emami et al. [28] studied the microbial consortium based on *Pseudomonas* strains, showing a significant increase in the growth of wheat plants. Jha and Saraf [29] reported that a multispecies consortium based on *Micrococcus* sp., *Acinetobacter calcoaceticus*, *Brevibacillus brevis*, and *Bacillus licheniformis* improved the yield of the *Jatropha curcas* plant via the ability of the consortium to produce indole-3-acetic acid (IAA), 1-aminocyclopropane-1-carboxylate (ACC) deaminase, and siderophores, and to solubilize inorganic phosphorus. In this context, the encapsulation of microbial cells in a polymeric matrix, with the aim of protecting the cells from the hostile environment, reduces microbial competition and gradually releases them, facilitating the rhizosphere colonization [30,31]. The materials used in encapsulation processes are alginate, starch, talc, and gelatin, as well as rice flour, skimmed milk, corn starch, and dextrose. However, algal polysaccharides—alginate, carrageenan, and agar—are the most widely used [32]. For instance, alginate and bentonite matrices offer advantages, since alginate favors greater cross-linking and the formation of spherical pearls, and bentonite is a low-cost matrix with photoprotection properties, which improves the mechanical resistance and the porosity of the pearls [33]. In addition, bentonite is used to improve the biosorption properties of potentially toxic elements, since clay minerals have cellular sorption and protection capacities to cope with different types of stress, thus improving the bioremediation and plant growth capabilities [34]. Based on the above, the aim of this study was to analyze the encapsulation of two consortia of metal(loid)-resistant bacteria in bentonite-enriched alginate beads and how these bacterial encapsulates influence physiological *C. quinoa* plant parameters. We used microbial resources obtained from the hyper-arid environment of Atacama Desert (northern Chile) and King George Island (Antarctica) because of the previous corroboration of their drought and metal tolerance, induced by the permanent water shortage and the usual geological high metal(loid) levels of soils. The main contribution of this work lies in the selection of the optimal matrix to immobilize living cells that support an improved plant physiological parameter. This research highlights the potential for creating low-cost microbial inoculants that could improve agricultural practices through bioencapsulation of bacterial consortia.

## 2. Materials and Methods

### 2.1. Microorganism

The bacterial consortia were previously isolated and formulated before being used in this study [13]. In detail, two consortia were employed: (i) consortium A, consisting of *Pseudomonas* sp. (GenBank accession OK235660) and *Bacillus* sp. (GenBank accession OK235661); and (ii) consortium B, composed of *Pseudomonas* sp. (GenBank accession OK235660) and *Bacillus* sp. (GenBank accession OK235659). The *Pseudomonas* strains were isolated from King George Island (62°02′00″ S 58°21′00″ W) in Antarctica and *Bacillus* strains were isolated from Atacama Desert (19°17′05″ S 69°23′29″ W). These bacterial strains were obtained from the microbial collection of the Bioremediation Laboratory at the University of La Frontera in Temuco, Chile.

### 2.2. Biosorption of Metal(loid)s of Rhizosphere Bacterial Consortia

Prior to the encapsulation, the biosorption capacity of consortia A and B was determined. The biosorption of Cu, Mn, and As was determined according to a previous study [35]. Experiments were performed in 50 mL of metal(loid) mix solution, based on the minimum inhibitory concentration of the consortia determined in a previous study [13]. In detail, metal(loid) mix I: 70, 234, and 97 mg L^−1^ of Cu(II), Mn(II) and As(V), respectively; and metal(loid) mix II: 200 mg L^−1^, 1000 mg L^−1^, and 50 mg L^−1^ of Cu(II), Mn(II), and As(V), respectively. Bacterial biomass was incubated in the metal(loid) solutions on an orbital shaker at 26 °C for 96 h at 100 rpm. The biomass was separated by centrifugation at 5000 rpm for 10 min and the supernatant was analyzed for residual metal(loid) concentration by inductively coupled plasma atomic emission spectroscopy (ICP-OES) (Thermo ICAPQ, Madrid, Spain). The amount of metal(loid) bound by the biosorbent was calculated as (1):

(1) $$Q=\frac{V\left(C_{i}-C_{f}\right)}{M}$$

where Q = metal(loid) ion uptake capacity (mg g^−1^); C_i_ = initial concentration of metal(loid)s (mg g^−1^); C*_f_* = final concentration of metal(loid)s in solution after the sorption analysis (mg g^−1^); M = dry weight of biosorbent (g); and V = solution volume (L). Additionally, metal(loid) uptake was also expressed as metal(loid) removal efficiency, given by (2):

(2) Metal(loid)s removal efficiency(%)=100(Ci−Cf)/Ci

### 2.3. Encapsulation

#### 2.3.1. Bacterial Encapsulation by Ionic Gelation

Bacterial consortia were encapsulated in alginate and bentonite microgels using an ionic gelation method [36]. Briefly, four encapsulation matrices were used: (1) sodium alginate solution 3% (*w*/*v*) and bentonite 2% (*w*/*v*); (2) sodium alginate solution 3% (*w*/*v*), bentonite 2% (*w*/*v*), and glycerol 3% (*w*/*v*); (3) sodium alginate solution 3% (*w*/*v*), bentonite 2% (*w*/*v*), and LB medium 2.5% (*w*/*v*); and (4) sodium alginate solution 3% (*w*/*v*), and molasses 3% (*w*/*v*). The matrices were sterilized and then cooled to room temperature. Each sterile solution (100 mL each) was mixed with 65 or 80 mL of ~10^8^ CFU mL^−1^ of cells. The polymer solution was shaken to distribute the cells throughout the mixture. Alginate beads were prepared aseptically using an encapsulator (Büchi B-390 R, Büchi Labortechnik AG, Flawil, Switzerland). After 2 h of stirring in 1.5% CaCl_2_, the resulting calcium alginate beads were collected by filtration and washed with sterile deionized water (200 mL). The beads were dried and stored at different temperatures for further analysis.

#### 2.3.2. Analysis of Drying Temperature and Microbial Viability of the Beads

To identify the most effective drying temperature, 10 g of wet beads was subjected to drying at temperatures of 25, 28, 30, and 35 °C using a drying oven (Zhicheng, ZFD-5140, Shanghai, China). The microbial viability of the dried beads was assessed by storing them at 4 and 24 °C for one year, with survival measurements taken every two months. To determine the optimal drying temperature and monitor the viability of the beads over time, samples weighing 0.1 g were ground in a sterile mortar with sterile 0.95% NaCl to extract the bacteria. The concentration of bacterial colony-forming units (CFU g^−1^) was determined by plating six serial dilutions on Luria–Bertani (LB) agar plates using the extended plate method. The plates were then incubated at 26 °C for two days before counting the colonies. The experiments were conducted in triplicate. Bacterial survival was determined using Equation (3) as previously described [37].

(3) $$Survivability={LOG}_{10}(N_{t}/N_{i})$$

where N_i_ is the number of viable cells at time zero and N_t_ is the number of viable cells after drying or viability treatments over time.

#### 2.3.3. Pearl Encapsulation Efficiency (EE)

The encapsulation efficiency of bacterial consortia was determined according to a previous study [38]. The beads were disintegrated in NaCl 0.95% and subsequently the entrapped viable bacteria were counted by the pour plate technique in LB agar. The EE was calculated according to Equation (4).

(4) $$EE(\%)=\frac{LOG\left[N\right]}{LOG\left[No\right]}\times 100$$

where *N* is the number of entrapped viable bacteria cells and *No* represents the free viable bacteria cells before encapsulation (CFU g^−1^).

#### 2.3.4. Size and Sphericity Factor

The size of the capsules was determined according to a previous study [39]. Briefly, 24.5-megapixel photographs were taken of approximately 100 capsules in a Petri dish with a millimeter paper in the bottom. The open-source software, ImageJ (https://imagej.net/ij/, accessed on 11 October 2024; version 1.54f) was used for analysis. The sphericity factor (SF) was then calculated using Equation (5):

(5) $$Sphericity\;factor\;(SF)=\frac{D_{max}-D_{per}}{D_{max}+D_{per}}$$

where *D_max_* is the largest diameter that passes through the centroid of the bead and *D_per_* is the diameter perpendicular to the previous one.

#### 2.3.5. Analysis of Bead Swelling

The swelling ratio (*SR*) of the microcapsules was determined according to a previous study [40]. The dried beads were immersed in a physiological saline solution (0.95%). After reaching the swelling equilibrium (48 h), the beads were taken out and weighed after careful blotting with tissue paper to remove surface water. The SR of beads was calculated according to Equation (6):

(6) $$SR\;(\%)=\frac{W_{t}-W_{d}}{W_{d}}*100$$

where W_d_ is the dry weight of the pearls and W_t_ is the weight of the pearls after reaching the swelling equilibrium. All the experiments were carried out six times (n = 6).

### 2.4. Greenhouse Experiment

#### 2.4.1. Soil Characteristics and Measurements

For this study, we utilized agricultural soil from Vilcún, Chile, which was collected at a 20 cm depth. Approximately 0.15 g of soil sample was weighed, sieved, and subsequently analyzed for Cu, As, and Mn levels using inductively coupled plasma atomic emission spectrometry (ICP-OES) [41]. The samples were prepared in accordance with ref. [42] and each was analyzed three times (Table 1).

#### 2.4.2. Experimental Design

The solutions of the metal(loid) mix were added to the soil two months before transplanting the seedlings. Each plant was inoculated with 1 g of beads (10^7^ UFC g^−1^). The experiments consisted of a three-factorial design. In our study, two matrices were selected for their ability to maintain bacterial viability over time. The inoculation of the microbial consortium (beads without inoculum, consortium A, or consortium B) and the supply of the metal(loid) mix (soil without the metal(loid) mix, mix I: 70 mg L^−1^, 234 mg L^−1^, and 97 mg L^−1^ of Cu(II), Mn(II), and As(V), respectively; or mix II: 200 mg L^−1^, 1000 mg L^−1^, and 50 mg L^−1^ of Cu(II), Mn(II), and As(V), respectively), and the dual and triple interactions were the main sources of variation, with a total of 18 treatments in quadruplicate (n = 6; N = 108).

#### 2.4.3. Growth Conditions and Measurements

To conduct the experiment, the *C. quinoa* Regalona Baer cultivar was utilized. The seeds were subjected to a superficial sterilization process involving a one-minute immersion in 90% ethanol, followed by a two-minute exposure to 10% sodium hypochlorite. Subsequently, they were rinsed using sterile deionized water. The germination process involved placing the seeds in a germination tray. Once the seedlings reached 30 days of age, each was then transplanted into pots, with a depth of 2 cm. These pots contained the alginate-encapsulated bacterial consortia, weighing 1 g, as well as the corresponding control setups. To ensure a suitable growing environment, the pots were filled with 635 g of sterilized soil. All the experimental units (pots) were placed in a bioclimatic chamber with supplementary light provided by Sylvania incandescent and cool-white lamps, 400 E m^−2^ s^−1^, 400–700 nm, with a 16/8 day/night cycle at 24/19 °C for 90 days. Throughout the experiment, the plants were regularly irrigated with distilled water, maintaining the soil moisture at a consistent level of 60% of its water retention capacity. At the time of harvest, the plants were divided into their root and shoot components.

Morphological variables were measured, including shoot length and diameter (cm), root dry weight (g), shoot dry weight (g), and flower length (cm). All measurements were conducted six times to ensure reliable results. Samples of leaves and roots were preserved at a temperature of −80 °C for subsequent analysis of photosynthetic pigments and lipid peroxidation.

Photosynthetic pigments were determined using the method described in a previous study [43]. Briefly, 0.1 g of fresh leaf sample was incubated at 65 °C for 30 min with 7 mL of dimethyl sulfoxide (DMSO). A quantity of 3 mL of DMSO was added to the sample. In the solution mixture, the contents of chlorophyll-a, chlorophyll-b, and total chlorophyll were spectrophotometrically analyzed at 645 and 663 nm, using DMSO as the blank.

Malondialdehyde (MDA) content was determined using the method described in a previous study [44]. Briefly, 0.15 g of fresh plant material from 6 individual leaves of each treatment was homogenized with 2 mL of 0.1% trichloroacetic acid in cold mortars, of which 1.8 mL was transferred to Eppendorf tubes. Subsequently, 0.5 mL of the supernatant was heated with 20% thiobarbituric acid + 0.5% tributyric acid in a thermoblock at 95 °C for 30 min. The sample was then quickly cooled on ice and centrifuged at 10,000× *g* for 10 min. The absorbance of the reaction product was spectrophotometrically read at 440, 532, and 600 nm. The MDA concentration was calculated using a molar extinction coefficient of 155 mM^−1^ cm^−1^.

The analysis of metal(loid) content in *C. quinoa* was conducted 90 days after the treatment commenced. *C. quinoa* leaves were harvested and then dried at 60 °C for 3 days and the Cu, Mn, and As content was assessed using ICP-OES analysis.

### 2.5. Statistical Analysis

The data analyses were carried out through factorial ANOVA depending on the type of experiment (encapsulation or greenhouse trays), considering specific factors and variables accordingly. We utilized two software tools: IBM SPSS Statistics (IBM Corp. Released 2021, Version 28.0; IBM Corp., Armonk, NY, USA) and R software version 4.2. In R, we harnessed several libraries and packages, including readr, dplyr, ggplot2, and corrplot, to facilitate data manipulation, visualization, and correlation analysis. To assess the statistical significance of our findings, we established a *p*-value < 0.05. To validate the statistical assumptions, we subjected the datasets to the Kolmogorov–Smirnov and the Shapiro–Wilk tests. In cases where substantial deviations from normality were detected, we applied a log_10_ transformation to address these deviations.

## 3. Results

### 3.1. Biosorption of Metal(loid)s in the Bacterial Consortia

**Figure 1:**
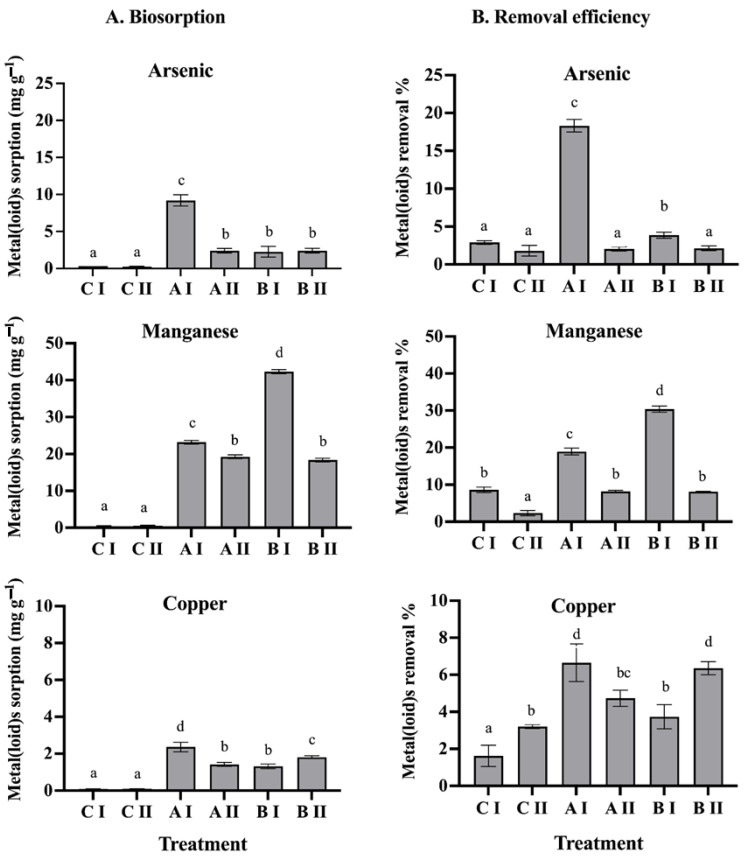
(**A**): Biosorption and (**B**): removal efficiency of the metal(loid)s by consortium A and consortium B. CI: metal(loid) mix I. CII: metal(loid) mix II. AI: metal(loid) mix I with consortium A. AII: metals(loid) mix II with consortium A. BI: metal(loid) mix I with consortium B. BII: metals(loid) mix II with consortium B. Different letters above bars indicate significant differences among treatments (*p* < 0.05, n = 6).

The biosorption capacity of consortium A (*Pseudomonas* sp., GenBank accession OK235660, and *Bacillus* sp., GenBank accession OK235661), and consortium B (*Pseudomonas* sp., GenBank accession OK235660 and *Bacillus* sp., GenBank accession OK235659) was evaluated in the presence of two metal(loid) mixes. Based on the amount adsorbed per unit mass of adsorbent, consortium A showed a significantly higher biosorption capacity, of 9.2 mg As(V) g^−1^, 23.2 mg Mn(II) g^−1^, and 2.3 mg Cu g^−1^ (Figure 1A). On the other hand, consortium B showed biosorption capacities of 1.2 mg As(V) g^−1^, 41.7 mg Mn(II) g^−1^, and 2.3 mg Cu g^−1^ (Figure 1A). Regarding the metal(loid) removal efficiency, consortium A showed the highest percentage of As(V) removal, of 18.3%, while consortium B showed the highest percentage of Mn(II) removal, of 31.4% in mix I. Furthermore, it was observed that both the Q and the RE% of the metals were significantly affected as the concentration of the metal(loid)s increased (see mix II). In consortium A, the removal efficiency for As(V) decreased to 2%, and in consortium B, it decreased by 8% for Mn(II) (Figure 1B).

### 3.2. Encapsulation Matrices 

A total of four encapsulation matrices were developed, primarily composed of 3% alginate and 2% bentonite. These matrices were further enriched with additional compounds, including glycerol, LB medium, and molasses. Encapsulation efficiencies ranging from 73% to 99% were achieved using initial bacterial cultures at 1.6 × 10^7^ to 9.8 × 10^8^ CFU mL^−1^. Among the four encapsulation matrices, matrix A1 showed the highest efficiency of 99% for both bacterial consortia. Matrices A2, A3, and A4 also displayed higher efficiencies of encapsulation, surpassing 94%, 89%, and 73%, respectively. The sphericity factor, which indicates the roundness of the beads, was found to be between 0.04 and 0.11. Matrices A1 and A3 exhibited the highest level of sphericity, with a value of 0.04, indicating that these matrices produced the most-spherical beads among the tested options. Additionally, the pearls demonstrated swelling ranging from 23% to 44%. Matrix A2 exhibited the highest swelling percentage, with values of 43% for consortium B and 44% for consortium A (Table 2).

### 3.3. Analysis of Drying Temperature and Analysis of Microbiological Viability of the Beads

#### 3.3.1. Drying Temperature Analysis

**Figure 2:**
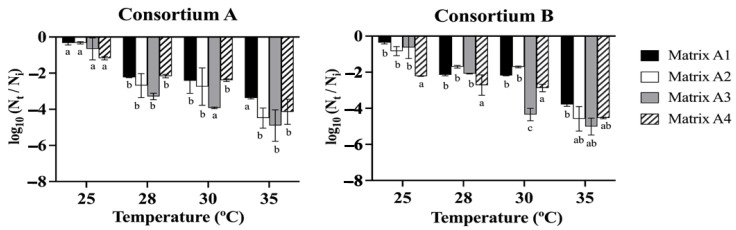
Survival of bacterial consortia A and B in different matrices. Matrix (A1 alginate 3% bentonite 2%), matrix A2 (alginate 3% bentonite 2% glycerol 3%), matrix A3 (alginate 3%, bentonite 2%, LB medium 2.5%), and matrix A4 (alginate 3% molasses 3%). The beads were dried at 25, 28, 30, and 35 °C for 24 h. N_t_ is the number of viable cells after drying, N_i_ is the number of viable cells at time zero. The initial cell count was approximately 10^9^ CFU g^−1^. The different letters indicate a statistically significant difference between the groups *p* < 0.05.

The data obtained from the drying treatments (Figure 2) indicated that the viability of consortia A and B was not significantly affected at a drying temperature of 25 °C, resulting in minimal loss of cell viability compared to the control of 10^8^ CFU g^−1^. Commonly, matrix A1, which consisted of 3% alginate and 2% bentonite, exhibited lower levels of cell viability loss in all treatment conditions. Furthermore, the results showed that there is a trend where the increase in the drying temperature significantly decreased the cell viability of all the matrices after exposure between 28 and 35 °C for 24 h. At a temperature of 28–35 °C, there were no significant differences in the cell viability between the matrices, except for matrix A3 (alginate 3%, bentonite 2%, and LB medium 2.5%), which presented a greater loss of viability, reaching up to 5.01 log cycles, approximately 10^3^ CFU g^−1^, followed by matrix A2 (alginate 3%, bentonite 2%, glycerol 3%) and matrix A4 (alginate 3%, molasses 3%), with losses of cell viability of 4.58 and 4.54 log cycles, approximately 10^4^ CFU g^−1^ (Figure 2).

#### 3.3.2. Feasibility Analysis in Bead

**Figure 3:**
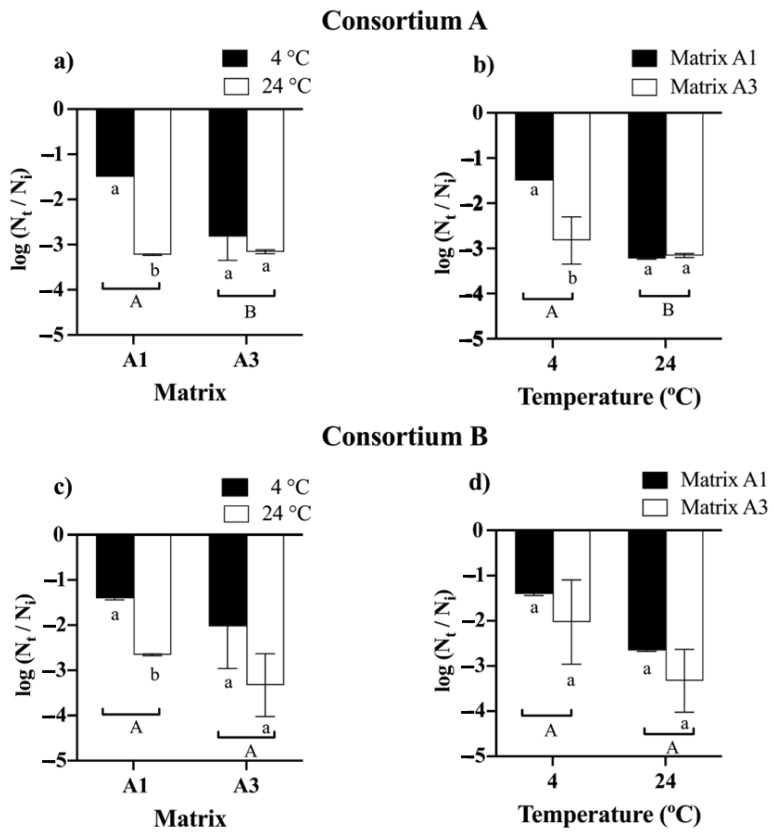
Viability of bacterial consortia A and B after 12 months of storage at 4 and 24 °C using different encapsulation matrices. Matrix A1 (3% alginate and 2% bentonite) and matrix A3 (3% alginate, 2% bentonite, and 2.5% LB medium). The initial cell count was approximately 10^8^ CFU g^−1^. Error bars indicate the standard deviation of 6 independent replicates. Lowercase letters indicate a significant difference between temperatures for each treatment and uppercase letters indicate a significant difference between matrices; *p* < 0.05.

The comparative analysis of bead storage demonstrated that time had a significant impact on cell viability. The initial average viability of the beads for consortia A and B was 10^8^ CFU g^−1^ (Figure 3). For consortia A and B, it was observed that beads formulated with matrices A2 and A4 experienced a complete loss of viability; for consortium A after 2 and 6 months (Figure S1), and for consortium B, at 4 and 8 months (Figure S2). Finally, at 12 months, for consortium A, it was observed that both the matrix and the temperature significantly influenced the viability of the beads. The best viability was obtained with matrix A1 and storage at 4 °C, resulting in a decrease in viability of approximately 1.5 log cycles or 10^6^ CFU g^−1^ (Figure 3a). On the other hand, for consortium B, it was observed that the matrices and the storage temperature did not have a significant effect on the viability of the beads. There was a decrease in the viability of 1.4 logarithmic cycles for matrix A1 and matrix A3 to 4 °C, resulting in a viability of approximately 10^6^ CFU g^−1^ (Figure 3c).

### 3.4. Greenhouse Experiment

#### 3.4.1. Morphological Characteristics of *Chenopodium quinoa*

**Figure 4:**
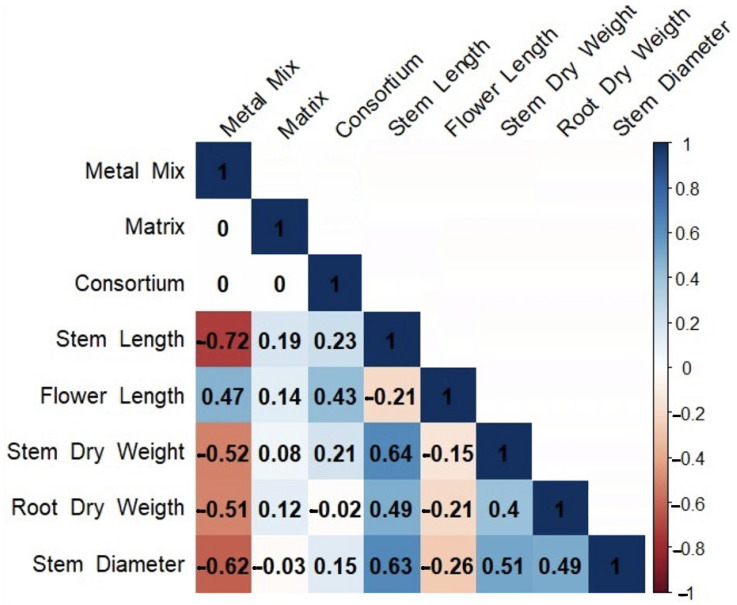
Spearman’s rank correlation analysis between consortium, matrix, metal(loid) mix, and morphological traits of *C. quinoa*. The cells are colored according to the correlation coefficient. Blue represents a significant positive correlation and red represents a significant negative correlation.

The use of the PGPB consortia on *C. quinoa* plants growing in soils contaminated with a metal(loid) mix showed that both consortia A and B significantly improved the plant growth (Table 3). Greater flower lengths were recorded for consortium A in matrix A3 and consortium B with matrix A3. On the other hand, regarding the dry weight of the shoot, it was found that consortium A with matrix A3 presented higher weights in the presence of the control soil mix, metal(loid) mix I, and metal(loid) mix II, respectively. In contrast, no significant differences in root dry weight were observed for any treatment compared to the control. Spearman’s correlation analysis evidenced a positive correlation between the consortia in almost all the variables studied. Moreover, a positive correlation was found between the metal(loid) mix and flower length. Furthermore, a negative correlation was observed between the metal(loid) mix and shoot length, stem diameter, shoot dry weight, and root dry weight, with strong negative effects on shoot length (Figure 4).

#### 3.4.2. Photosynthetic Pigments

**Figure 5:**
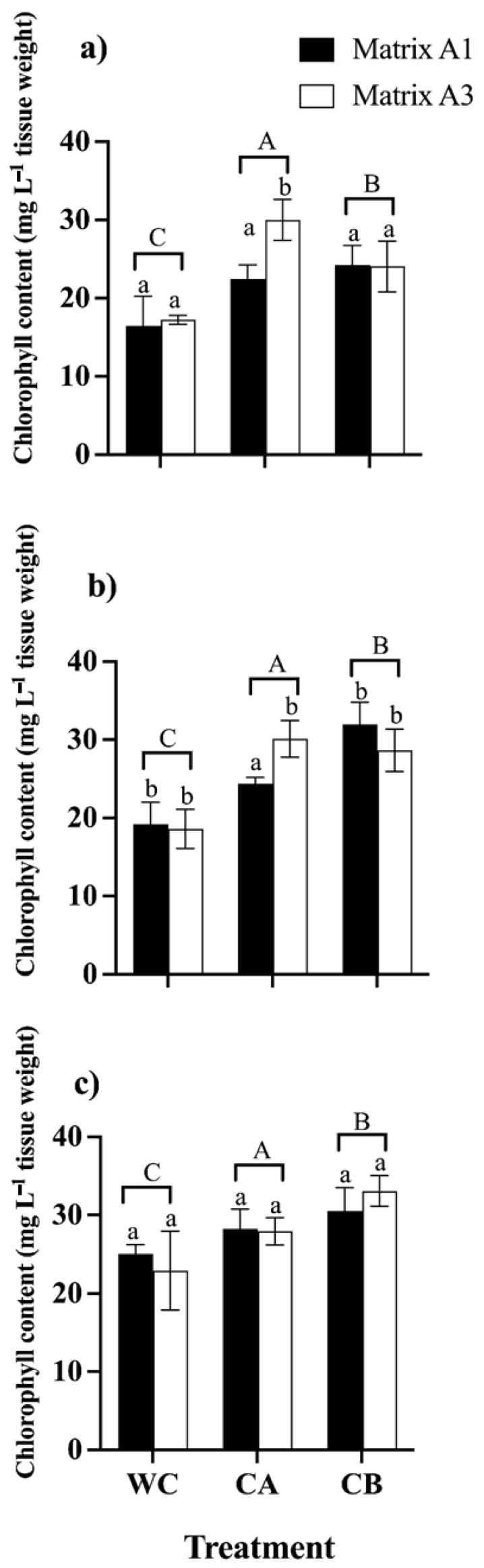
Total chlorophyll content in *C. quinoa* plants WC: without consortium, CA: with consortium A, and CB: with consortium B; matrix A1 (3% alginate and 2% bentonite) and matrix A3 (3% alginate, 2% bentonite, and 2.5% LB medium) in different concentrations of metal(loid)s. (**a**) Control: soil without the metal(loid) mix; (**b**) metal(loid) mix I, and (**c**) metal(loid) mix II. Error bars indicate the standard deviation of 6 independent replicates. Lower-case letters indicate a significant difference between matrices for each treatment and upper-case letters indicate a significant difference between treatments *p* < 0.05.

The leaf chlorophyll content was analyzed to investigate the influence of consortia A and B and the effect of matrices A1 and A3 on the photosynthetic efficiency of *C. quinoa* plants in the presence of metal(loid)s (Figure 5). The introduction of consortia A and B substantially enhanced the total chlorophyll content in the presence of metal(loid) mix stress. In detail, plants inoculated with consortium B displayed the highest content of total chlorophyll (24.01 mg L^−1^, 31.9 mg L^−1^, 33.29 mg L^−1^) when exposed to the mixture of control, metal(loid) mix I, and metal(loid) mix II, respectively. Finally, we concluded that matrices A1 and A3 in general do not exert an effect on the consortia and the synthesis of photosynthetic pigments when the plants are subjected to high concentrations of metal(loid)s (Figure 5).

#### 3.4.3. Lipid Peroxidation Assay

**Figure 6:**
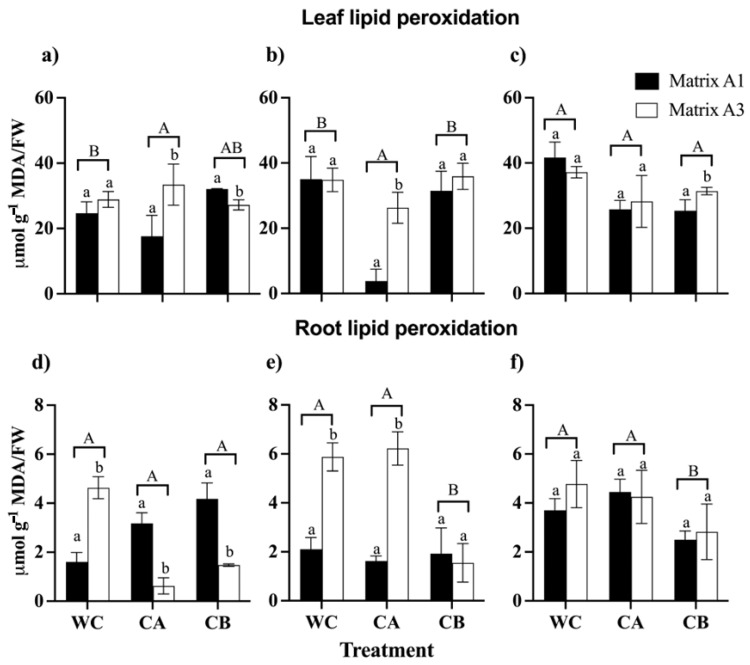
Malondialdehyde (MDA) content in leaves and roots of *C. quinoa* plants. WC: without consortium, CA: with consortium A and CB: with consortium B; Matrix A1 (3% alginate and 2% bentonite) and matrix A3 (3% alginate, 2% bentonite, and 2.5% LB medium) in different concentrations of metal(loid)s. (**a**,**d**) Control: soil without the metal(loid) mix; (**b**,**e**) metal(loid) mix I and (**c**,**f**) metal(loid) mix II. Error bars indicate the standard deviation of 6 independent replicates. Lower-case letters indicate a significant difference between matrices for each treatment and upper-case letters indicate a significant difference between treatments *p* < 0.05.

The results demonstrated that there was an increase in lipid peroxidation in the leaves and roots of *C. quinoa* due to the rise in concentrations of metal(loid)s (Figure 6). Notably, leaves exhibited a more pronounced induction of MDA compared to roots. Regarding the MDA content in leaves, the study revealed that consortium A in matrix A1 significantly reduced the MDA content. Specifically, the MDA values were 17.7 µmol g^−1^/FW in the control treatment and 3.8 µmol g^−1^/FW in the metal(loid) mix I treatment. On the contrary, in the leaves, consortium B and the matrices did not show a significant effect on the MDA content in any treatment with a metal(loid) mix. Furthermore, it is important to mention that in the treatment with metal(loid) mix II, there were no significant differences between the consortia and the matrices regarding the control group (Figure 6c). On the contrary, in the roots the results indicated that consortium A did not have a significant effect on the reduction in MDA in any of the treatments compared to the control. Moreover, consortium B in matrices A1 and A3 had a significant impact on reducing MDA levels. In both the metal(loid) mix I and metal(loid) mix II treatments, the MDA values were lower than 2.1 µmol g^−1^ FW and 2.8 µmol g^−1^ FW, respectively (Figure 6e,f). In general, it was found that matrices A1 and A3 exhibited significant differences for the same consortium.

#### 3.4.4. Chemical Analysis of Plant Tissues

The chemical analysis of metal(loid) contents in *C. quinoa* leaves indicated that higher concentrations of Cu and Mn in the soil were associated with an increase in the contents of these metal(loid)s in the leaves. Furthermore, consortium A in matrix A3 enhanced the uptake of Cu by 59.9 mg kg^−1^ in the metal(loid) mix II treatment. On the contrary, consortium B matrix A1 appears to reduce the absorption of Cu to 32.1 mg kg^−1^ in metal(loid) mix II, and consortium B matrix A3 reduced the absorption of Mn to 171.9 mg kg^−1^ in the control. Finally, the absorption of As in the leaves of *C. quinoa* was not detected in any of the treatments (Table 4).

## 4. Discussion

Soil plays a key role in food safety; therefore, in a scenario of increasing metal(loid) contamination, the use of encapsulated inoculants emerges as a sustainable alternative for plant production [20,45,46]. In our study, (i) consortium A, consisting of *Pseudomonas* sp. (GenBank accession OK235660) and *Bacillus* sp. (GenBank accession OK235661); and (ii) consortium B, composed of *Pseudomonas* sp. (GenBank accession OK235660) and *Bacillus* sp. (GenBank accession OK235659) had arsenic removal rates of 18% and manganese removal rates of 31%, respectively. This suggests their bioremediation capacity, which is representative of the adaptation of tolerant bacteria to metal toxicity [47]. However, this removal rate decreased to about 2% and 8%, respectively, when the consortia were exposed to higher concentrations of metals in metal(loid) mix II. This could be due to a higher concentration; the consortia have difficulties in maintaining an efficient removal rate due to additional toxicity leading to sorption inhibition or saturation of the adsorption sites. Excessive metal ions tend to block the available active sites, thus preventing the subsequent ions from entering the adsorption active sites. Therefore, the rate of sorption capacity slowed down and the removal rate decreased in the case of arsenic concentration [48,49]. Similar results were reported by Rizvi et al. [50], where the bacterial strains *Pseudomonas aeruginosa* and *Bacillus subtilis* decreased absorption by 6% when the Cu concentration increased from 25 to 400 mg Kg^−1^, a similar trend followed for cadmium and chromium.

Bioencapsulation in agriculture offers key benefits over other bioformulations, such as trapping high cell concentrations, improving microbial viability, maintaining the efficacy of plant-growth-promoting properties, and controlled, long-term release for effective plant growth promotion and root colonization [51]. However, there are still certain limitations, including that the encapsulation matrices show different degradation rates against abiotic factors such as high temperatures and, on the other hand, the impact on the native microbiome in different soil types [52]. To prolong the useful life of the consortia, they were encapsulated in different matrices, mainly composed of alginate and bentonite. The results showed that the encapsulation efficiency ranged between 73% and 99%, with the highest efficiency observed in the matrix of alginate and bentonite (matrix A1). However, the removal of bentonite resulted in a significant loss of efficiency, which is consistent with previous observations of Batista et al. [33] and He et al. [53]. This suggests that the removal of bentonite from the encapsulation matrix can significantly affect the efficiency of the process of encapsulation. This is supported by studies conducted by Kragović et al. [54] and Zhou & Sun [55]. In addition, Zhang et al. [56] showed that hydrogen bonding and electrostatic attraction between alginate and bentonite allow for the formation of a compact structure. In this sense, the properties of sodium alginate depend on the composition of its linear chains of α-L-glucuronic acid and β-D-mannuronic acid [57]. The structure and its capacity to bind bacteria are largely influenced by the sequence of these monomers. Additionally, sodium alginate supports biofilm formation and promotes the adhesion and colonization of microorganisms, which favors microbial survival [58]. On the other hand, the incorporation of bentonite into sodium alginate enhances the solid contents and addresses the limitations of stand-alone alginate beads, such as low porosity [53]. This addition also boosts mechanical strength by increasing viscosity and stability. As a result, the release of bacteria is controlled and sustained, as bentonite reduces the corrosion of the calcium alginate matrix, thereby enhancing the stability of the encapsulated bacteria over time and ensuring their long-term efficacy. This supports the results found in our study, where the most efficient encapsulation and sphericity were found in matrices containing alginate and bentonite.

The size of the beads is an important characteristic to consider for their use in experiments under field conditions [59]. In our study, the values of diameters and surface factors (SFs) allowed an effective beneficial interaction between the microorganisms encapsulated in matrices A1 and A3 with *C. quinoa* plants, due to their diameters being less than 1.7 mm and SFs being less than 0.04. Studies by Szopa et al. [60] and Piornos et al. [61] suggest that smaller and more spherical beads can have important advantages in bioremediation processes by improving the interaction with plants and facilitating the homogeneity of microorganism release, which are key aspects for the success of consortia used as bioinoculants.

The percentage of swelling of the beads was closely related to the release of the encapsulated microorganisms and showed a continuous release of the microorganisms (with a swelling percentage greater than 23.5% in 48 h). Previous studies have highlighted that the release kinetics tend to decrease at higher concentrations of bentonite, which ensures the stability of the microorganisms over time [33]. This trend suggests an interesting strategy to explore different concentrations of bentonite, as this material has a high affinity for metal(loid)s and allows for a controlled release of microorganisms [62]. The results obtained support the importance of adjusting bentonite concentrations to achieve a controlled and sustained release of microorganisms, thus maximizing their effectiveness in bioremediation. 

On the other hand, the consortia were significantly affected at drying temperatures of 28 °C and 35 °C, which can be attributed to the fact that the strains comprising the consortia have optimal growth temperatures between 4 °C and 28 °C, and the optimal growth temperature of *Pseudomonas* sp. is between 4 °C and 24 °C [13]. In addition, a minimal loss of viability observed at 24 °C can be attributed to the drying process of the beads, as it has been shown that a significant percentage of bacterial cells die during bead dehydration [63]. Here, we detected the best survival rates after drying and storage in the A1 matrix at 4 °C after 12 months, which maintained concentrations of 10^6^ CFU g^−1^, which is the essential threshold to produce a positive response in plants; for example, the threshold for *Azospirillum brasilense*, which promotes plant growth, is 10^6^–10^7^ cells per plant [30]. The combination of alginate and bentonite has been widely studied for the immobilization of microbial cells due to the protective barrier and moisture control properties it provides to the cells during the drying process [64,65,66]. Similar results were previously reported [63], where the survival rate of encapsulated cells was 1 year at 4 °C. On the other hand, He et al. [53] determined the cell viability of *Raoultella planticola* Rs-2 in bentonite and alginate matrices after 6 months and reported a survival rate of 89%. Therefore, matrices formed by alginate and bentonite could be used as effective sources of carbohydrates and protective agents. However, it is interesting to note that the use of glycerol and molasses for long-term storage of microorganisms is not recommended due to the low viability found [67]. On the other hand, traditional methods such as peats, broth cultures, and clays are widely used as microbial inoculants in agricultural practices, but these techniques do not provide optimal protective environments and cell viability in comparison with our bioencapsulation of bacterial cells. In this sense, Schoebitz et al. [68] concluded that traditional microorganism inoculants should be stored at room temperature, since their shelf life is very short, and temperature oscillations, which considerably decrease their viability, should be avoided. Instead, bioencapsulation (using alginate) provides a niche where bacteria are protected from soil stress. In this way, the polymeric matrix ensures a slow release of the bacterial consortia into the soil, being more effective over time [60].

Regarding the morphological parameters of *C. quinoa*, it was found that the bacterial consortia significantly improved the growth characteristics of the treated plants. However, the growth of plants inoculated and treated with the metal(loid) mix was lower (Table 3). Consortium A in matrix A3 showed the best performance, which is related to the reported PGP properties (indoleacetic acid, ACC deaminase, phosphate solubilization, among others) reported in a previous study [13]. These consortia not only promoted plant growth, but also stimulated chlorophyll synthesis and decreased malondialdehyde (MDA) production. However, increased MDA levels in leaves indicate oxidative stress in plants due to metal accumulation [69]. This accumulation of metal(loid)s in *C. quinoa* leaves is consistent with previous research by Haseeb et al. [70] and Muhammad et al. [71], who highlighted the capacity of this species to accumulate high concentrations of Cd, Pb, and Cu. High levels of MDA correlate with chemical analysis of the leaves, which revealed elevated concentrations of metalloids. These findings suggest that although bacterial consortia enhance plant growth, metal(loid) accumulation generates oxidative stress, highlighting the importance of mitigating this accumulation to maximize the benefits of consortia on *C. quinoa* growth.

Consortium B appeared to reduce the uptake of Cu to 32 mg kg^−1^ and Mn to 172 mg kg^−1^. However, although consortium B reduced Cu and Mn accumulation in *C. quinoa* tissues in metal(loid) mix II, plant Cu levels still exceeded recommended levels (30 mg kg^−1^) [72]. In addition, As was not detected in the leaves of *C. quinoa*, even though the plants grew at As concentrations of 97 mg L^−1^, which are higher than those reported in soils where *C. quinoa* is usually cropped [73]. However, there is evidence that *C. quinoa* bioaccumulates As in the shoot and seed even at concentrations <40 mg L^−1^ [74]. Ultimately, consideration of these factors is essential to assess the potential risks associated with metal(loid) uptake by crops and to establish guidelines or regulations to ensure food safety and environmental health. In this sense, Shilev et al. [75] concluded that the incorporation of bacterial consortia composed of the genera *Pseudomonas* and *Bacillus* reduces metal uptake (Pb, Cd, and Zn) in *Spinacea oleracea* plants. These mechanisms can be attributed to an increase in the production of ACC deaminase activity, siderophores, and indole-3-acetic acid.

Considering the microbial communities in the rhizosphere, the development of consortia with more than one bacterial species, without an antagonistic behavior between them, can produce or induce changes in the composition and functioning of the soil microbiome [76]. In effect, in our study, the consortia triggered plant defense mechanisms that reduced the negative effects of metals and can be used to promote plant growth and soil bioremediation [25,77]. Although bacterial consortia have shown beneficial and long-lasting impacts on the soil microbiome [78], their indirect effects are still not fully understood.

## 5. Conclusions

The current study determined that encapsulating bacterial consortium A and consortium B improved the survival rate of bacteria and extended the viability period up to 12 months at 4 °C, particularly with matrix A1 composed of 3% alginate and 2% bentonite. Furthermore, inoculating *Chenopodium quinoa* plants with encapsulated consortia improved growth under metal(loid) stress, promoted chlorophyll synthesis, and reduced lipid peroxidation. Consortium B appeared to decrease Cu and Mn absorption; however, concentrations of these metals exceeded recommended values for consumption. Thus, the use of *C. quinoa* leaves growing in soils with metal contamination levels ≥ metal(loid) mix II is not advised. Finally, these findings recommend the use of encapsulated bacterial consortia A and B as bioinoculants to promote the growth of *C. quinoa* in non-contaminated soils or soils that do not exceed the minimum inhibitory concentration of the strains.

## Figures and Tables

**Table 1:** Chemical analysis of the soil samples from Vilcún, Chile.

	Initial Concentrations
Arsenic (As) mg Kg^−1^	<0.01
Copper (Cu) mg Kg^−1^	126 ± 2.99
Manganese (Mn) mg Kg^−1^	2202 ± 36.37
pH (H_2_O)	5.58

**Table 2:** Parameters of consortia A and B encapsulation by ionic gelation (n = 6).

Matrix	Encapsulating Agent	Consortia	Initial Culture Total CFU	Encapsulation Efficiency (%)	Sphericity Factor	Size (mm)	Beads Swelling %
A1	Alginate 3% Bentonite 2%	A	(9.2 ± 0.7) × 10^7^	99	0.04 ± 0.01	0.7 ± 0.05	32.7 ± 6.4
		B	(9.8 ± 0.7) × 10^7^	99	0.04 ± 0.03	0.7 ± 0.04	33.7 ± 4.4
A2	Alginate 3% Bentonite 2% Glycerol 3%	A	(1.6 ± 0.3) × 10^8^	94	0.11 ± 0.02	1.4 ± 0.09	44.3 ± 6.3
		B	(2.6 ± 0.3) × 10^8^	95	0.10 ± 0.02	1.6 ± 0.05	43.5 ± 2.2
A3	Alginate 3% Bentonite 2% LB medium 2.5%	A	(2.3 ± 0.7) × 10^8^	89	0.04 ± 0.01	0.7 ± 0.05	38.6 ± 1.8
		B	(2.2 ± 0.7) × 10^8^	90	0.04 ± 0.01	0.7 ± 0.05	35.3 ± 1.2
A4	Alginate 3% Molasses 3%	A	(4.6 ± 0.3) × 10^8^	73	0.05 ± 0.01	0.9 ± 0.14	23.5 ± 1.0
		B	(4.8 ± 0.3) × 10^8^	74	0.05 ± 0.02	0.9 ± 0.04	24.1 ± 0.4

**Table 3:** Evaluation of the morphological parameters of *C. quinoa* plants in contaminated soils. Data represent the mean ± standard deviation (n = 6). The letters represent significant differences between formulations for each metal(loid) treatment (*p* < 0.05).

Metal(loid) Treatments	Formulation	Shoot Length (cm)	Flower Length (cm)	Root Dry Weight (g)	Stem Diameter (cm)	Shoot Dry Weight (g)
Control	Matrix A1	32.2 ± 0.6 a	2.4 ± 0.7 a	0.7 ± 0.1 a	0.33 ± 0.2 a	5.2 ± 0.1 a
	Matrix A3	33.3 ± 0.7 a	2.5 ± 0.4 a	0.8 ± 0.1 a	0.36 ± 0.4 a	5.1 ± 0.1 a
	Consortium A in Matrix A1	37.7 ± 0.8 b	3.4 ± 0.5 bc	0.7 ± 0.1 a	0.33 ± 0.2 a	6.8 ± 0.4 b
	Consortium A in Matrix A3	39.6 ± 0.9 c	3.9 ± 0.8 bc	0.9 ± 0.1 a	0.37 ± 0.4 a	7.6 ± 0.6 c
	Consortium B in Matrix A1	37.1 ± 0.7 b	3.2 ± 0.5 b	0.8 ± 0.1 a	0.34 ± 0.3 a	5.7 ± 0.3 a
	Consortium B in Matrix A3	34.7 ± 0.8 ab	4.2 ± 0.5 c	0.6 ± 0.1 a	0.31 ± 0.2 a	5.2 ± 0.1 a
Metal(loid) mix I	Matrix A1	30.7 ± 0.5 b	2.3 ± 0.3 a	0.3 ± 0.1 a	0.26 ± 0.5 a	5.7 ± 0.8 a
	Matrix A3	28.5 ± 0.5 a	3.5 ± 0.3 bc	0.4 ± 0.1 ab	0.22 ± 0.5 a	5.4 ± 0.3 a
	Consortium A in Matrix A1	31.8 ± 0.6 b	3.5 ± 0.2 bc	0.5 ± 0.2 b	0.35 ± 0.3 a	6.8 ± 0.4 b
	Consortium A in Matrix A3	36.2 ± 0.7 d	4.1 ± 0.6 cd	0.5 ± 0.2 b	0.35 ± 0.5 a	7.5 ± 0.4 b
	Consortium B in Matrix A1	34.1 ± 0.7 c	4.6 ± 0.5 d	0.4 ± 0.2 ab	0.30 ± 0.5 a	5.9 ± 0.5 a
	Consortium B in Matrix A3	32.7 ± 0.7 b	4.0 ± 0.2 cd	0.6 ± 0.2 b	0.31 ± 0.2 a	5.9 ± 0.4 a
Metal(loid) mix II	Matrix A1	17.3 ± 0.6 a	4.1 ± 0.7 a	0.4 ± 0.1 a	0.19 ± 0.5 a	2.0 ± 0.2 a
	Matrix A3	17.1 ± 0.7 a	3.8 ± 0.6 a	0.6 ± 0.1 a	0.23 ± 0.5 ab	2.2 ± 0.5 a
	Consortium A in Matrix A1	26.9 ± 0.8 b	4.7 ± 0.8 a	0.4 ± 0.1 a	0.28 ± 0.2 b	2.7 ± 0.7 ab
	Consortium A in Matrix A3	30.1 ± 0.3 c	4.9 ± 0.6 a	0.8 ± 0.1 a	0.25 ± 0.1 ab	3.4 ± 0.8 bc
	Consortium B in Matrix A1	29.2 ± 0.7 c	5.6 ± 0.1 a	0.3 ± 0.0 a	0.29 ± 0.5 b	4.2 ± 0.7 d
	Consortium B in Matrix A3	28.9 ± 0.8 c	4.9 ± 0.5 a	0.4 ± 0.1 a	0.27 ± 0.4 b	3.2 ± 0.4 bc

Control: Soil without the metal(loid) mix, metal(loid) mix I: (70 mg L^−1^, 234 mg L^−1^, and 97 mg L^−1^ of Cu(II), Mn(II), and As (V)), respectively, and metal(loid) mix II: 200 mg L^−1^, 1000 mg L^−1^, and 50 mg L^−1^ of Cu(II), Mn(II), and As(V), respectively.

**Table 4:** Cu, As, and Mn contents in *C. quinoa* leaves under different metal(loid) mix concentrations. Control: soil without the metal(loid) mix, metal(loid) mix I: 70 mg L^−1^, 234 mg L^−1^, and 97 mg L^−1^ of Cu, Mn(II), and As(V), respectively, and metal(loid) mix II: 200 mg L^−1^, 1000 mg L^−1^, and 50 mg L^−1^ of Cu, Mn(II), and As(V), respectively. Matrix A1 (3% alginate and 2% bentonite) and matrix A3 (3% alginate, 2% bentonite, and 2.5% LB medium).

Formulation	Metal Mixes mg L^−1^	mg Cu kg^−1^	mg As kg^−1^	mg Mn kg^−1^
Consortium A in Matrix A1	Control	7.75	ND	213.70
	Metal(loid) Mix I	8.61	ND	354.57
	Metal(loid) Mix II	50.72	ND	430.71
Consortium A in Matrix A3	Control	7.89	ND	239.36
	Metal(loid) Mix I	6.38	ND	294.16
	Metal(loid) Mix II	59.87	ND	305.33
Consortium B in Matrix A1	Control	6.57	ND	258.67
	Metal(loid) Mix I	7.14	ND	339.44
	Metal(loid) Mix II	32.13	ND	322.53
Consortium B in Matrix A3	Control	6.11	ND	171.92
	Metal(loid) Mix I	9.71	ND	331.97
	Metal(loid) Mix II	38.91	ND	357.73
Matrix A1	Control	9.65	ND	291.65
	Metal(loid) Mix I	9.65	ND	282.13
	Metal(loid) Mix II	39.74	ND	335.62
Matrix A3	Control	7.63	ND	260.46
	Metal(loid) Mix I	6.58	ND	375.45
	Metal(loid) Mix II	43.36	ND	336.52

ND: Not detected.

## Data Availability

The original contributions presented in the study are included in the article/Supplementary Material, further inquiries can be directed to the corresponding author.

## Supplementary Materials

The following supporting information can be downloaded at: https://www.mdpi.com/article/10.3390/microorganisms12102066/s1, Figure S1: Viability of bacterial consortium A (*Pseudomonas* sp. and *Bacillus* sp.) after 12 months of storage at 4 °C and 24 °C using different encapsulation matrices. Matrix A1 (alginate 3% bentonite 2%), Matrix A2 (alginate 3% bentonite 2% glycerol 3%), Matrix A3 (alginate 3% bentonite 2% LB medium 2.5%) and Matrix A4 (alginate 3% molasses 3%). The initial cell count was approximately 10^8^ CFU g^−1^. Error bars indicate standard deviation of 4 independent replicates. Lowercase letters indicate a significant difference between temperatures for each treatment and uppercase letters indicate a significant difference between matrices *p* < 0.05. Figure S2: Viability of bacterial consortium B (*Pseudomonas* sp. and *Bacillus* sp.) after 12 months of storage at 4 °C and 24 °C using different encapsulation matrices. Matrix A1 (alginate 3% bentonite 2%), Matrix A2 (alginate 3% bentonite 2% glycerol 3%), Matrix A3 (alginate 3% bentonite 2% LB medium 2.5%) and Matrix A4 (alginate 3% molasses 3%). The initial cell count was approximately 10^8^ CFU g^−1^. Error bars indicate standard deviation of 4 independent replicates. Lowercase letters indicate a significant difference between temperatures for each treatment and uppercase letters indicate a significant difference between matrices *p* < 0.05.
