# Correction: Alvarado et al. Alginate–Bentonite Encapsulation of Extremophillic Bacterial Consortia Enhances *Chenopodium quinoa* Tolerance to Metal Stress. *Microorganisms* 2024, *12*, 2066

**DOI:** 10.3390/microorganisms12112356

**Published:** 2024-11-19

**Authors:** Roxana Alvarado, Cesar Arriagada-Escamilla, Javier Ortiz, Reinaldo Campos-Vargas, Pablo Cornejo

**Affiliations:** 1 Laboratorio Biorremediación, Departamento de Ciencias Forestales, Facultad de Ciencias Agropecuarias y Medioambiente, Universidad de La Frontera, Temuco 4811230, Chile; roxanalvaradoab@gmail.com (R.A.); javier.ortiz@ufrontera.cl (J.O.); 2 Programa de Doctorado en Ciencias de Recursos Naturales, Universidad de La Frontera, Temuco 4811230, Chile; 3 Scientific and Technological Bioresource Nucleus (BIOREN), Universidad de La Frontera, Temuco 4811230, Chile; 4 Center for Postharvest Studies, Faculty of Agricultural Sciences, Universidad de Chile, Santiago 8820808, Chile; reinaldocampos@uchile.cl; 5 Centro Regional de Investigación e Innovación para la Sostenibilidad de la Agricultura y los Territorios Rurales, CERES, Pontificia Universidad Católica de Valparaíso, La Palma, Quillota 2260000, Chile; pcornejo@centroceres.cl

In the original publication [1], there was a mistake in the order of authors. The content was directly derived from Roxana Alvarado’s original Ph.D. thesis, which he completed in full at the University of La Frontera. The authors believe that the authorship should accurately reflect his primary contribution. The correct format appears below:

Roxana Alvarado ^1,2,†^, Cesar Arriagada-Escamilla ^1,^*^,†^, Javier Ortiz ^1,3^, Reinaldo Campos-Vargas ^4^ and Pablo Cornejo ^5^.

The corrected Author Contributions statement appears here. The authors state that the scientific conclusions are unaffected. This correction was approved by the Academic Editor. The original publication has also been updated.
