# Supplementary material for: Alginate–Bentonite Encapsulation of Extremophillic Bacterial Consortia Enhances Chenopodium quinoa Tolerance to Metal Stress
Source: Microorganisms. 2024 Oct 15;12(10):2066. doi: 10.3390/microorganisms12102066 (PMC11509983; doi:10.3390/microorganisms12102066)
Supplement: Supplementary file 1 [file microorganisms-12-02066-s001.zip › microorganisms-3255487-supplementary.pdf]

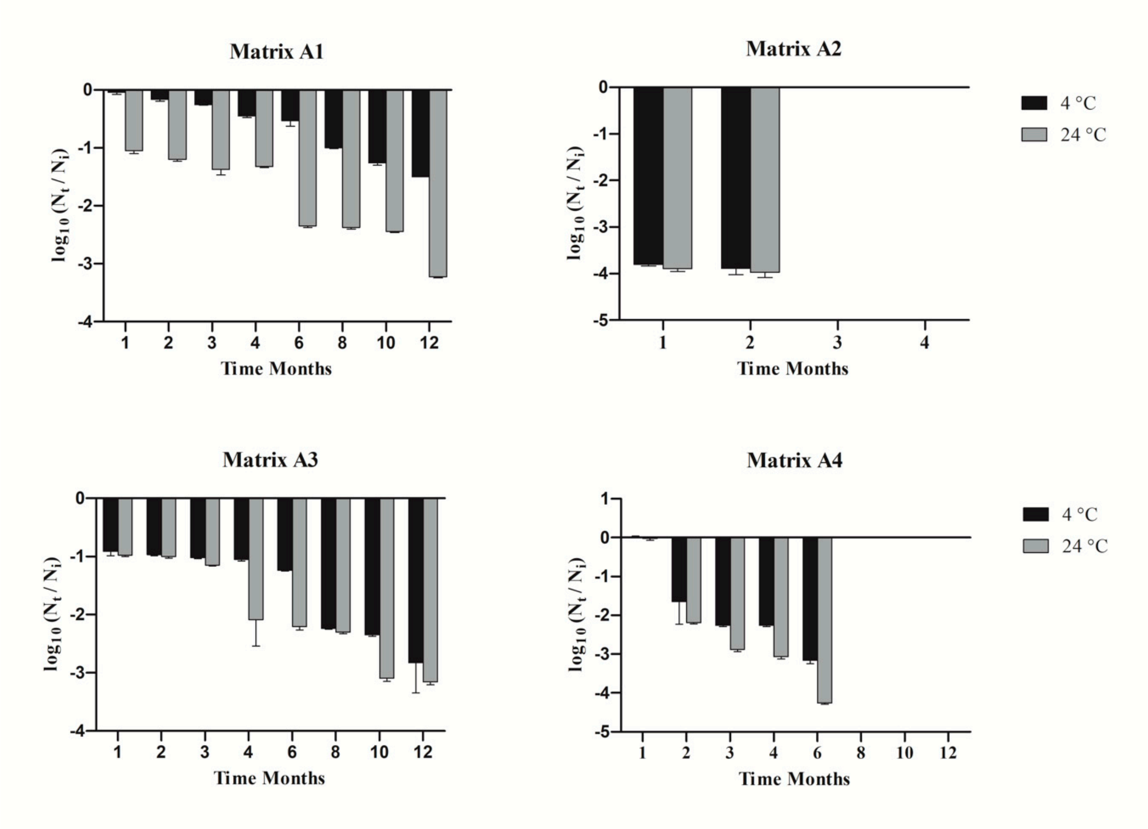

Supplementary Figure S1 Viability of bacterial consortium A (*Pseudomonas* sp. and *Bacillus* sp.) after 12 months of storage at 4 °C and 24 °C using different encapsulation matrices. Matrix A1 (alginate 3% bentonite 2%), Matrix A2 (alginate 3% bentonite 2% glycerol 3%), Matrix A3 (alginate 3% bentonite 2% LB medium 2.5%) and Matrix A4 (alginate 3% molasses 3%). The initial cell count was approximately 10^8 CFU g^{-1}. Error bars indicate standard deviation of 4 independent replicates. Lowercase letters indicate a significant difference between temperatures for each treatment and uppercase letters indicate a significant difference between matrices $P < 0.05$.

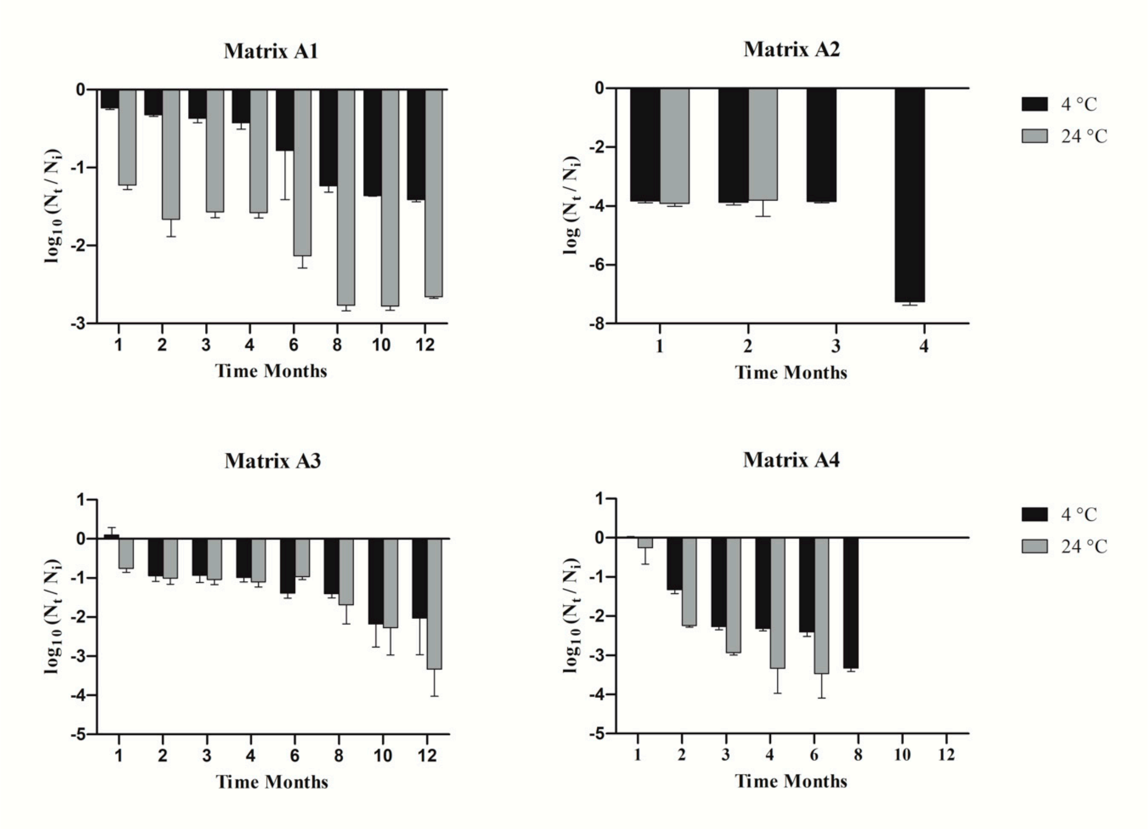

Supplementary Figure S2 Viability of bacterial consortium B (*Pseudomonas* sp. and *Bacillus* sp.) after 12 months of storage at 4 °C and 24 °C using different encapsulation matrices. Matrix A1 (alginate 3% bentonite 2%), Matrix A2 (alginate 3% bentonite 2% glycerol 3%), Matrix A3 (alginate 3% bentonite 2% LB medium 2.5%) and Matrix A4 (alginate 3% molasses 3%). The initial cell count was approximately 10^8 CFU g^{-1}. Error bars indicate standard deviation of 4 independent replicates. Lowercase letters indicate a significant difference between temperatures for each treatment and uppercase letters indicate a significant difference between matrices $P < 0.05$.
